# Diagnostic Performance of Contrast Enhanced Pulmonary Computed Tomography Angiography for the Detection of Angioinvasive Pulmonary Aspergillosis in Immunocompromised Patients

**DOI:** 10.1038/s41598-017-04470-6

**Published:** 2017-06-30

**Authors:** C. Henzler, T. Henzler, D. Buchheidt, John W. Nance, C. A. Weis, R. Vogelmann, U. Benck, T. Viergutz, T. Becher, T. Boch, S. A. Klein, D. Heidenreich, L. Pilz, M. Meyer, P. M. Deckert, W.-K. Hofmann, S. O. Schoenberg, M. Reinwald

**Affiliations:** 1 Institute of Clinical Radiology and Nuclear Medicine, University Medical Center Mannheim, Medical Faculty Mannheim, Heidelberg University, Heidelberg, Germany; 2 Department of Hematology and Oncology, University Medical Center Mannheim, Medical Faculty Mannheim, Heidelberg University, Heidelberg, Germany; 3 Department of Radiology, Medical University of South Carolina, Charleston, SC USA; 4 Department of Pathology, University Medical Center Mannheim, Medical Faculty Mannheim, Heidelberg University, Heidelberg, Germany; 5 Department of Gastroenterology and Infectious Diseases, University Medical Center Mannheim, Medical Faculty Mannheim, Heidelberg University, Heidelberg, Germany; 6 Department of Nephrology, University Medical Center Mannheim, Medical Faculty Mannheim, Heidelberg University, Heidelberg, Germany; 7 Department of Anesthesiology and Intensive Care Medicine, University Medical Center Mannheim, Medical Faculty Mannheim, Heidelberg University, Heidelberg, Germany; 8 Department of Cardiology, University Medical Center Mannheim, Medical Faculty Mannheim, Heidelberg University, Heidelberg, Germany; 9 University Medical Center Mannheim, Medical Faculty Mannheim, Heidelberg University, Heidelberg, Germany; 10 Department of Hematology and Oncology, Medical University of Brandenburg (MHB) Theodor Fontane, Brandenburg an der Havel, Germany

## Abstract

Invasive pulmonary aspergillosis (IPA) is one of the major complications in immunocompromised patients. The mainstay of diagnostic imaging is non-enhanced chest-computed-tomography (CT), for which various non-specific signs for IPA have been described. However, contrast-enhanced CT pulmonary angiography (CTPA) has shown promising results, as the vessel occlusion sign (VOS) seems to be more sensitive and specific for IPA in hematologic patients. The aim of this study was to evaluate the diagnostic accuracy of CTPA in a larger cohort including non-hematologic immunocompromised patients. CTPA studies of 78 consecutive immunocompromised patients with proven/probable IPA were analyzed. 45 immunocompromised patients without IPA served as a control group. Diagnostic performance of CTPA-detected VOS and of radiological signs that do not require contrast-media were analyzed. Of 12 evaluable radiological signs, five were found to be significantly associated with IPA. The VOS showed the highest diagnostic performance with a sensitivity of 0.94, specificity of 0.71 and a diagnostic odds-ratio of 36.8. Regression analysis revealed the two strongest independent radiological predictors for IPA to be the VOS and the halo sign. The VOS is highly suggestive for IPA in immunocompromised patients in general. Thus, contrast-enhanced CTPA superior over non-contrast_enhanced chest-CT in patients with suspected IPA.

## Introduction

Invasive pulmonary aspergillosis (IPA) is a severe, life-threatening complication in immunocompromised patients, contributing significantly to morbidity and mortality^1^. Complicating matters, the definitive diagnosis of IPA remains challenging, and the currently available diagnostic tools provide suboptimal accuracy^2^.

The *European Organization of Treatment and Research of Cancer* and the *Mycosis Study Group* (EORTC/MSG) defined strict clinical, radiological and mycological criteria for the diagnosis of IPA in immunocompromised patients^3^. Modified criteria have been proposed for patients treated in the intensive care unit (ICU)^4^, which have shown improved diagnostic accuracy in this population. However, in clinical routine, the EORTC/MSG criteria for “proven IPA” can rarely be achieved, as this requires direct evidence of fungal pathogens by histopathology or culture from sterile specimens. “Probable IPA” requires less rigorous mycology criteria, including positive culture from non-sterile specimens (e.g. Bronchoalveolar lavage (BAL)) or biomarkers such as galactomannan (GM) or beta-D-glucan (BDG). Patients without positive mycological evidence but with clinical suspicion of IPA are deemed to have “possible IPA,” and according to the EORTC/MSC consensus criteria, imaging is the diagnostic backbone for these patients.

Several CT patterns are associated with IPA. Perhaps the most prominent, the halo sign, is seen when nodular or consolidative opacities are surrounded by ground-glass attenuation ^5^. In hematologic patients, refractory to broad-spectrum antibacterial antibiotics, the halo sign yields a positive predictive value for IPA of about 70–80%. Unfortunately, the halo sign is not specific for IPA since other infectious pathogens, such as *Pseudomonas* and *Cryptococcus*, as well as neoplastic and autoinflammatory conditions can also produce this pattern^5^. In addition, the diagnostic value of the halo sign in non-hematologic immunocompromised patients has been questioned^5^. The relatively low specificity of non-contrast enhanced chest CT alone was demonstrated in a retrospective study where only 50% of patients with suspected IPA based on non-contrast enhanced CT findings actually suffered from IPA as determined by tissue or post-mortem autopsy findings^6^.

Given the essential role and current limitations of CT, improvements in its diagnostic performance in IPA should be actively pursued. Clinical guidelines recommend performing a low dose chest CT without contrast media in immunocompromised patients suspected of having IPA. However, Sonnet *et al*. have shown that CT pulmonary angiography (CTPA) may increase the diagnostic potential in this situation: In a trial encompassing 12 proven/probable invasive mould disease (IMD) patients, the authors demonstrated that IPA patients had CTPA findings consistent with interruption of arterial vessels^7^. This so called “vessel occlusion sign” (VOS) reflects the angioinvasive growth pattern of *Aspergillus* spp. and *Mucor* spp. that can be observed *in vivo*. The same group recently published data on an expanded patient cohort in a pivotal single-centre trial, evaluating 100 suspected hematologic IPA patients, of whom 46 patients suffered from proven or probable IPA. This study revealed the VOS as the strongest diagnostic radiological finding for IPA in immunocompromised patients with hematologic malignancies^8^. The current study evaluates the accuracy of CTPA in diagnosing IPA in a broader cohort of immunocompromised patients, including both those with hematologic malignancies and other causes.

## Patients and Methods

### Patients

This retrospective single-centre study was approved by our institutional review board and complies with both the Declaration of Helsinki and the Health Insurance Portability and Accountability Act (HIPAA). Due to the retrospective nature of the study protocol, the local ethical committee waived the need for written informed consent.

We retrospectively analysed patients’ electronic records and hospital charts from 1999 to 2014 and identified 455 immunocompromised patients with proven/probable IPA based on the 2008 EORTC/MSG consensus definitions. Of these 455 patients, 78 received a CTPA study for the diagnostic work-up during an infectious episode of suspected IPA and were therefore included in this study. Patient characteristics are summarized in Table 1.

**Table 1:** Patient characteristics of proven/probable IPA cases.

Characteristics	Proven/probable IPA* (n = 78)
Age, years, median (range)	60.0 years (2–84)
Gender (female/male)	27/51
Underlying disease	
AML	20
ALL	2
NHL	14
COPD	5
MPN	1
MDS	1
Aplastic Anemia	1
Hodgkins’ disease	2
Solid organ transplant recipients	6
Solid Tumor	11
Other^§^	15

*according to 2008 EORTC/MSG criteria for non-ICU and BLOT criteria for ICU patients

IPA: invasive pulmonary aspergillosis; AML: acute myeloid leukemia; ALL: acute lymphoblastic leukemia; NHL: Non-Hodgkin’s-Lymphoma; COPD: chronic obstructive pulmonary disease; MDS: myelodysplastic syndrome; MPN: myeloproliferative neoplasia;

§Consisting of polymyalgia rheumatica, goodpasture syndrome; HIV infection with low CD4-cell count < 250/µl, granulomatous polyangiitis, rheumatoid arthritis, Crohn’s disease, vasculitis, liver cirrhosis.

Forty-five equally immunocompromised patients with pulmonary infiltrates but no evidence of IPA based on the EORTC/MSG criteria served as a control group (NoIPA group). In the majority of patients, extended efforts were taken to identify the infectious organism, e.g. by performing bronchoscopy with bronchoalveolar lavage (BAL).

### Definitions for Classification

Immunocompromised patients were classified based on the 2008 EORTC/MSG consensus criteria^3^. ICU patients who did not meet the criteria for immunosuppression by the EORTC/MSG but had evidence of *Aspergillus* involvement were classified according to the criteria proposed by Blot *et al*., which have been shown to perform better in identifying IPA in ICU patients^4^.

### Radiological examinations

CTPA studies were performed either on a 16-slice MDCT system (SOMATOM Emotion, Siemens Healthineers, Forchheim, Germany) or on a 1^st^ generation 2 × 32 slice dual-source CT system (SOMATOM Definition, Siemens Healthineers, Forchheim, Germany). The contrast injection protocol included the injection of 80 ml of Iomeprol (Imeron 400, Bracco, Milan, Italy) with a flow-rate of 4 ml/s via an antecubital vein followed by a 40 ml saline chaser using the same injection rate. The bolus tracking method was used to determine the scan start. The scan automatically began 5 s after a threshold of 100 HU was reached within a region of interest placed within the pulmonary trunk, with scanning performed in a cranio-caudal direction.

Images were re-analysed for this study in a consensus reading by two chest radiologists (CH, TH) blinded to the patients’ diagnoses, clinical courses, and complications. The definition of the observed patterns followed the glossary of terms for thoracic imaging proposed by the Fleischner Society^9^. The following radiological patterns were evaluated:

- *nodular opacities*
- *halo sign*
- *cavern*
- *air-crescent sign*
- *internal low attenuation*
- *pleural effusion*
- *ground-glass opacity*
- *reversed halo sign*
- *tree-in-bud sign*
- *infarct-shaped consolidations*
- *crazy paving*
- *vessel occlusion sign* (VOS)

According to Sonnet *et al*., vascular occlusion was defined as a vessel interruption at the border of a focal lesion without extension of the vessel into or out of the lesion^7^. The presence of the VOS was only evaluated in lesions with a diameter ≥ 10 mm (or >12 mm in the peripheral lung).

### Timing of CTPA

As radiological patterns of IPA have been shown to change over the course of the infection and the halo sign being an early indicator of IPA^10^, the time of CTPA relative to the start of the infectious episode (as defined by the beginning of the increase of systemic inflammation markers [C-reactive protein or procalcitonine]) was evaluated in a subgroup of patients.

### Evaluation of renal function

Baseline and follow-up serum creatinine values were recorded to evaluate potential nephrotoxicity caused by contrast agent administration. Significant contrast-agent mediated nephrotoxicity was defined according to Stacul *et al*.^11^. Briefly, contrast medium-induced nephropathy (CIN) was defined as an increase in serum creatinine by ≥ 25% within 3 days after contrast agent administration in the absence of plausible alternative etiologies.

### Statistical analysis

Diagnostic performance of CTPA was calculated by comparing all proven/probable IPA patients (n = 78) with the No-IPA (n = 45) control population.

Sensitivity analysis was performed; odds ratios and 95%-confidence interval of these ratios were calculated. For discrete variables Fishers’ exact test and the χ²-test were used, as indicated.

For the logistic regression analysis, the leading response (dichotomous) variable was proven/probable IPA using a binary logit model with Fisher’s scoring as an optimization technique. The entry probability into the logistic regression model was set to 0.45 and the probability of stay was 0.55. Model building was started with all 12 diagnostic imaging variables. Goodness of fit was tested by deviance, Pearsons’ test, and the Hosmer-Lemeshow test. Level of significance was set to 0.05. Statistical software SAS 9.4 (SAS Institute Inc., Cary, NC, USA) were used for descriptive statistics, sensitivity analysis, and logistic regression and for the latter also LogXact software (Cytel Studio, Version 9.00, Cytel Inc. Pune, USA).

## Results

### Patient characteristics

Characteristics of the 78 immunocompromised patients with proven/probable IPA and an available CTPA study are summarized in Table 1. Among all patients with proven/probable IPA, 41 had an underlying hematological disease, whereas the remaining 37 were immunocompromised due to other conditions, such as solid-organ-transplantation, chronic obstructive pulmonary disease (COPD) with intensive systemic steroid treatment, or autoimmune diseases such as granulomatosis with polyangiitis (formerly Wegener’s granulomatosis). All of these patients met the EORTC or Blot criteria based on intensity of underlying or iatrogenic immunosuppression. Based on microbiological results obtained from BAL, sputum culture, respiratory specimen or blood culture, 42/45 patients in the immunocompromised control cohort were found to have an infectious disease other than aspergillosis or mucormycosis; no etiological pathogen could be identified in the remaining three patients. Twenty-two percent of patients had been treated with antifungal therapy (AFT) prior to performance of CTPA defined as receiving > 1 daily dosage mould-active AFT.

### Occurrence of different CT signs in proven/probable IPA vs. NoIPA cases

Table 2 summarizes the frequency of all evaluated CT parameters for the IPA and NoIPA group. The nodular configuration of infiltrates was the most frequent radiological sign in 77/78 proven/probable IPA patients (99%). However, it was also frequently positive in the NoIPA group (73%). VOS was the second most prevalent radiological pattern in 61/78 (78%) proven/probable IPA patients, followed by the halo sign in 56/78 (72%), infarct-shaped lesions in 51/78 (65%) and the air-crescent sign in 34/78 (44%) patients.

**Table 2:** Diagnostic performance of different radiological patterns in IPA and controls.

Radiological pattern	Proven/probable IPA (n (%))	No IPA (n (%))	Sensitivity	Specificity	PLR	NLR	Odds ratio (CI)	p-value*
Nodule	77 (99%)	33 (73%)	0.99	0.27	1.35	0.05	28.00 (3.50–224.21)	<0.001
Halo sign	56 (72%)	14 (31%)	0.72	0.69	2.31	0.41	5.64 (2.53–12.56)	<0.001
Cavern	20 (26%)	9 (20%)	0.26	0.80	1.28	0.93	1.38 (0.57–3.36)	0.517
Air crescent sign	34 (44%)	10 (22%)	0.44	0.78	1.96	0.73	2.71 (1.18–6.22)	0.020
Pleural effusion	32 (41%)	18 (40%)	0.41	0.6	1.1	0.98	1.04 (0.49–2.20)	0.911**
Ground-glass opacity	16 (21%)	16 (36%)	0.21	0.64	0.58	1.23	0.47 (0.21–1.06)	0.088
Tree-in-bud	22 (28%)	16 (36%)	0.28	0.64	0.79	1.11	0.71 (0.33–1.56)	0.423
Internal low attenuation	32 (41%)	10 (22%)	0.41	0.78	1.85	0.76	2.44 (1.06–5.61)	0.048
Reversed halo	0 (0%)	0 (0%)	—	—	—	—	—	—
Vessel occlusion sign	61 (78%)	4 (9%)	0.78	0.91	8.80	0.24	36.78 (11.54–117.19)	<0.001
Infarct-shaped consolidation	51 (65%)	23 (51%)	0.65	0.49	1.28	0.71	1.81 (0.86–3.82)	0.131
Crazy paving	8 (10%)	3 (7%)	0.10	0.93	1.47	0.97	1.52 (0.38–6.07)	0.745

IPA: invasive pulmonary aspergillosis; PLR: positive likelihood ratio; NLR: negative likelihood ratio; CI: confidence interval;

*probability value of the exact Fisher test (p-value),

**χ²-test (p-value).

For the control group, nodular infiltrate patterns were observed in 33/45 patients in (73%). VOS was observed in 4/45 (9%), the halo sign in 14/45 (31%), infarct-shaped lesions in 23/45 (51%), and the air-crescent sign in 10/45 (22%).

Altogether, five radiological patterns were associated with IPA in a statistically significant manner: The halo sign, the VOS, internal low attenuation, the air-crescent sign and the nodule pattern (all p < 0.05). Detailed diagnostic performance of the different radiological signs is displayed in Table 2.

### Frequency of VOS or halo sign in hematological and non-hematological conditions

**Figure 1:**
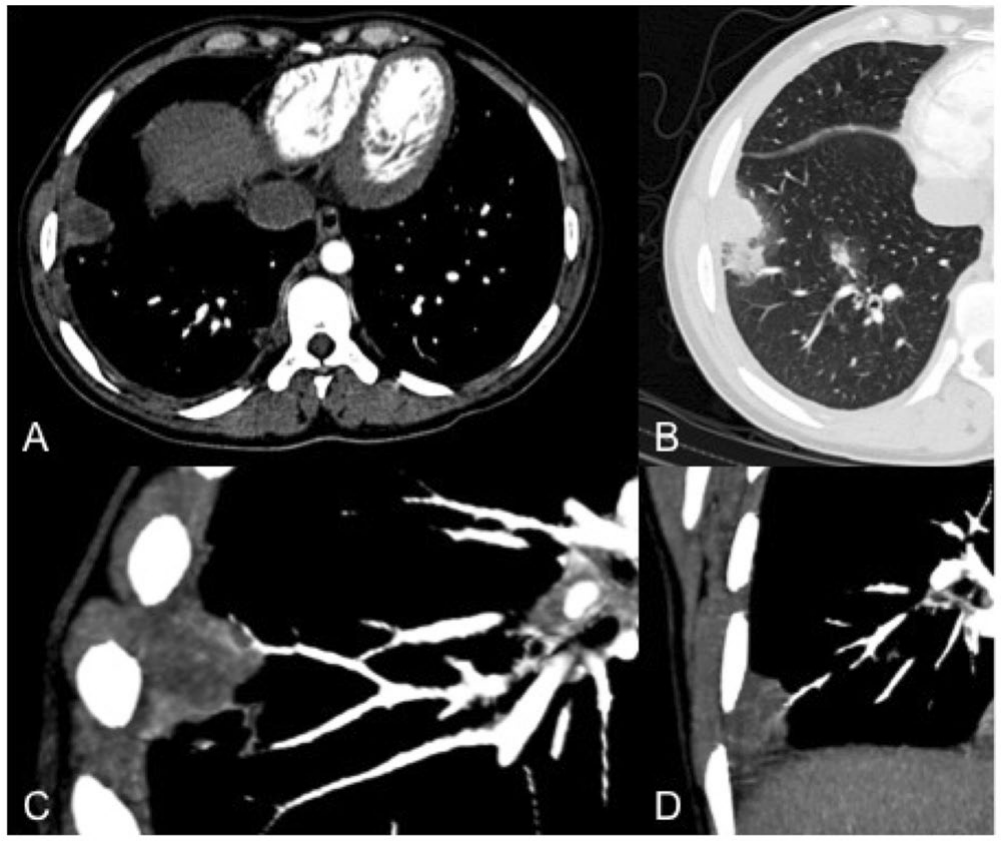
Computed tomography pulmonary angiography (CTPA) study of a 29-year-old male, immunocompromised patient. The infarct shaped subpleural consolidation showed central low attenuation (**A**) with a surrounding peripheral halo sign (**B**). CTPA showed vessel interruption within the lesion (**C**,**D**).

**Figure 2:**
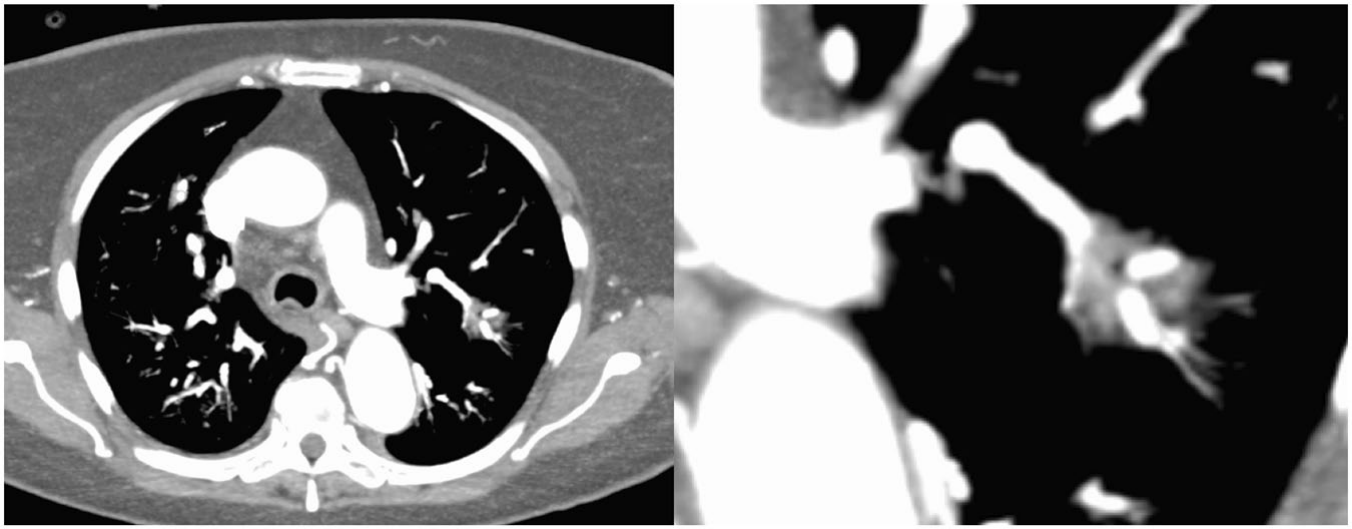
Overview (left) and close-up (right) computed tomography pulmonary angiography images of a 53-year-old immunocompromised patient due to kidney transplantation. The images demonstrate the vessel interruption within the sub-solid lesion.

We performed a subanalysis to evaluate whether the prevalence of VOS differs in immunocompromised patients with hematological disorders versus those without. VOS was seen in 33/41 (80%) patients with hematologic disorders and in 28/37 (76%) patients without hematologic disorders (no statistical difference; p > 0.6). Figures 1 and 2 display examples of VOS in immunocompromised patients with hematologic and non-hematologic conditions.

There was also no significant statistical difference in the prevalence of the halo sign between groups, seen in 32/41 (78%) patients with hematologic disorders and 24/37 (65%) patients without hematological disorders, although the difference was more pronounced (p > 0.2).

### Diagnostic performance of VOS in proven/probable IPA vs. NoIPA cases

VOS detected by CTPA was observed in 61/78 proven/probable IPA cases (78%), whereas it was only observed in 4/45 NoIPA cases (9%), leading to a sensitivity of 0.78, specificity of 0.91, positive likelihood ratio (PLR) of 8.8, and negative liklehood ratio (NLR) of 0.24. The diagnostic odds ratio (DOR) for VOS was thus 36.78 (95%CI 11.54–117.19).

Four patients in the control group showed a positive VOS: One NoIPA patient suffered from COPD with chronic systemic steroid treatment; however, steroid dose was not high enough to meet EORTC host criteria. In addition to the observed VOS, the patient showed infiltrate patterns typical for IPA, a positive halo sign, and positive blood Galactomannan (GM) as mycological evidence. The second VOS-positive NoIPA case was an ICU patient with multiorgan failure after polytrauma, positive GM in blood, and pulmonary embolism, possibly explaining the observed VOS. The third VOS-positive NoIPA patient also suffered from COPD and showed typical infiltrate patterns (including halo sign) with positive GM from blood and BAL. Due to insufficient host criteria; however, this patient was classified as NoIPA. The fourth VOS-positive NoIPA patient suffered from abscessing pneumonia and had a positive halo sign and positive blood GM. However, immunocompromising conditions were not strong enough according to EORTC/Blot criteria; accordingly, the patient was classified as NoIPA.

When comparing the diagnostic performance of the different radiological patterns, the VOS sign was found to be the strongest of all evaluated radiological signs in the detection of IPA. Table 2 summarizes the diagnostic performance of the different radiological signs.

### Frequency of VOS or halo sign in neutropenic and non-neutropenic conditions

Alltogether 37/78 Patients were neutropenic while 41/78 patients were non-neutropenic but immunosuppressed due to other conditions. In neutropenic patients VOS was prevalent in 31/37 patients while for non-neutropenic VOS showed positivity in 30/41 patients. This lead to a sensitivity of 84%, a specificity of 80%, a PLR of 4.2, an NLR of 0.2 and a DOR of 21 in neutropenic patients. For non-neutropenic patients a sensitivity of 73%, a specificity of 93%, a PLR of 9.8, an NLR of 0.3 and a DOR of 33.6 was observed.

### Diagnostic performance of VOS and halo sign relative to the start of the infection

Median time to CT was 7 days (range 0–41 days) for patients with proven/probable IPA wheareas it was 6 days (range 0–55 days) for patients with NoIPA. Statistical comparison between both proven/probable IPA vs. No IPA patients showed no significant difference between both groups (p > 0.92; Mann-Whitney-U test). As the halo sign has been shown to be an early indicator of IPA, subanalysis of patients who received the CT-scan+/−2 days relative to start of the infectious episode was performed: In these patients positivity for the halo sign was found in 9/17 proven/probable IPA patients (53%), while the VOS was positive in 15/17 of these patients (93%). Statistical analysis found the VOS to be significantly more frequent compared to the halo sign (p < 0.028; Mann-Whitney-U test).

### Influence of Antifungal treatment on VOS frequency

The vessel occlusion sign was detected in 86% of patients without mould-active agents prior to CTPA while positivity of VOS could be demonstrated for 73% of patients receiving AFT prior to performance of CTPA. This difference was not found to be statistically significant (p > 0.31; Mann-Whitney-U test). Four patients had received more than one mould-active antifungal agent prior to CTPA (two patients had liposomal Amphotericin B and Caspofungin, one patient had caspofungin and voriconazole and the last patient had liposomal Amphothericin B and Voriconazole); in all of these four patients VOS was positive.

### Statistical Evaluation and logistic regression model

Of the twelve evaluated radiological patterns, five radiological signs were found to be significantly associated with IPA: The VOS, the halo sign, the air crescent sign, the internal low attenuation sign, and the nodular configuration. Each showed a capacity of dichotomization between IPA and NoIPA patients with a p-value of <0.05.

The vessel occlusion sign (VOS), with the superior diagnostic odds ratio, served as the best discriminator between IPA and NoIPA.

### Stepwise logistic regression model

A logistic regression analysis was performed to check for independent association of these radiological signs, which often occur concurrently in the clinical setting. A stable model was built using the following seven variables which were entered into a stepwise procedure: *VOS, halo sign, crazy paving, nodules, infarct shaped infiltrate, cavern* and the *air-crescent sign*. Goodness of fit test showed a p-value of 0.77 in the the Hosmer-Lemeshow test, indicating that the model fits. A sum score model (adding up all 7 dichotomous variables) was built and seven observations were read. Goodness of fit test (deviance and Pearsons’ test) was given.

**Figure 3:**
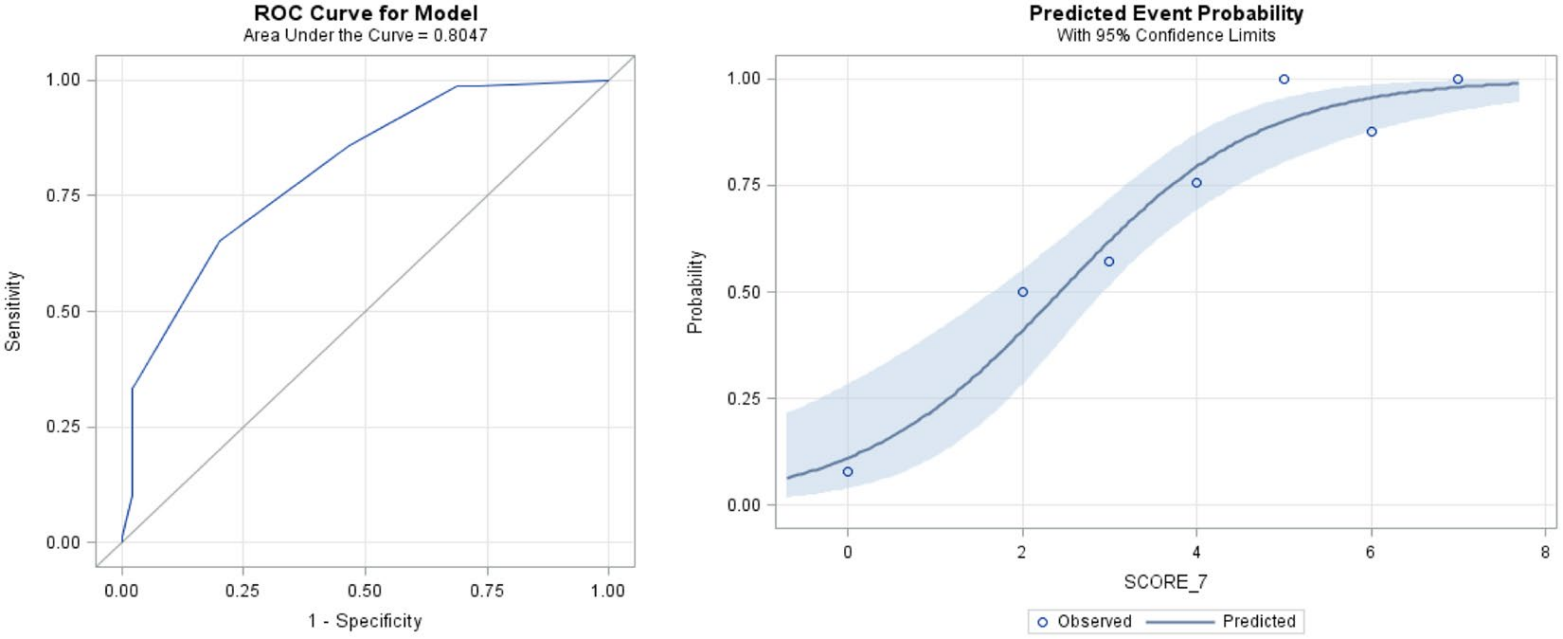
ROC analysis for the model with seven variables (VOS; halo sign, crazy paving, nodules, infarct shaped infiltrate, cavern and air-crescent sign) with an area under curve (AUC) of 0.805. The image on the right shows predicted event probability and the bands of 95%-confidence limits for the model with the seven variables.

The Receiver Operating Characteristic (ROC) analysis using these seven radiological signs is displayed in Fig. 3. The ROC had a steep initial increase and an area under the curve (AUC) of 0.805, suggesting a good positive diagnostic value. Removing the VOS from the model lead to a distinct decrease of AUC from 0.805 to 0.705, underlining the diagnostic placevalue of the VOS.

The logistic regression analysis revealed VOS and the halo sign as the two strongest independent radiological predictors for proven/probable IPA, with the VOS showing the most significant score (Chi-Square score 52.93; p < 0.0001, χ²-test).

### Safety of CTPA regarding nephrotoxicity

Median serum creatinine was 0.90 mg/dl (range 0.29–7.12 mg/dl) before CTPA (baseline) and 0.95 mg/dl (range 0.34–6.2 mg/dl) 48–72 hours after contrast agent exposure. Only two patients experienced an increase in serum creatinine of more than 25%, both however staying within the normal range, indicating a very tolerable rate of acute kidney injury according to published CIN criteria^11^.

## Discussion

Our study demonstrates that VOS as observed on CTPA examinations is superior to classic CT signs observed in non-contrast enhanced studies to diagnose invasive pulmonary aspergillosis in immunocompromised patients. This result held true regardless of the hematological or non-hematological origin of their condition and timing of the CT scan. As had been reported previously, we could not detect a significant influence of underlying antifungal therapy/prophylaxis on VOS performance. In addition, the use of intravenously injected contrast medium did not lead to nephrotoxicity in this patient cohort. This finding is in accordance to recent studies that questioned the harmfulness of intravenously injected contrast material even in patients with known kidney disease^12^.

IPA is a frequent but difficult diagnosis in severely immunocompromised patients^13^. Only histological examination and/or positive culture results from sterile sites provide definitive proof of IPA according to the latest EORTC/MSG consensus definitions^3^. Microbiological tests such as cultures or even biomarkers are often negative, especially under standard prophylactic or empiric antifungal medication^14, 15^. While this is often implemented as a routine clinical strategy, basing the diagnosis of IPA on non-contrast enhanced chest CT criteria alone is highly questionable. Only 50% of patients diagnosed with IPA by conventional CT criteria were found to truly harbor invasive fungal disease in microbiological examinations after surgical resection^6^. Other pulmonary infectious diseases can show similar radiological patterns^16^. At the same time, the hallmark halo sign^5, 17^ is of only short duration and thus easy to miss^10^, leading to a real-life sensitivity of about 0.8 in other studies and our own data^5^ so a considerable proportion of patients with IPA will go undiagnosed. Newer radiological techniques such as positron emitting tomography^18^ or magnetic resonance imaging^19^ are currently under evaluation for this diagnosis, but these tend to be less available and more laborious, time-consuming, and expensive.

By contrast, CTPA would provide an easily available improvement of the currently recommended imaging standard, and its non-invasiveness as opposed to bronchoscopy represents a clear advantage in immunocompromised patients who often are in critical clinical condition and at increased bleeding risk, prohibiting invasive diagnostic procedures. Moreover, as early diagnosis and early initiation of therapy improve survival in IPA patients^20^, the constant availability of CTPA in most institutions allows for fast and direct therapeutic decision-making, whereas bronchoscopy and subsequent pathological or microbiological examination as well as laboratory diagnostics of biomarkers can delay diagnosis for several days^21^.

As different radiological patterns often appear concurrently, a logistic regression analysis to further elucidate the independent diagnostic value of each radiological sign was performed and revealed that VOS independently was superior to the halo sign.

These findings of superior diagnostic performance of the contrast-enhanced VOS are in accordance with those of Stanzani *et al*. who initially proposed the use of CTPA in immunocompromised patients with hematologic disorders and suspected IPA^22^.

In addition to the former study, however, we broadened our cohort and for the first time included immunocompromised patients with conditions other than hematologic diseases.

It has been proposed that the prevalence of radiological patterns might be different depending on the disorder causing the reduced immunocompetence. In particular, hematologic malignancies were rather supposed to be associated with the halo sign compared to other conditions^23^. We did not find a significant difference between these groups for either VOS or the halo sign, although the difference in sensitivity between VOS and the halo sign was more pronounced in the non-hematological group. Furthermore we compared the diagnostic performance of VOS in neutropenic and non-neutropenic patients and could clearly underline its clinical usefulness even in non-neutropenic patients.

A concern in using iodine-based contrast media, especially in critically ill patients, is the risk of acute nephrotoxicity. In this study however, only few patients experienced CIN, and these even stayed within the normal kreatinine range, indicating that CTPA is safe in this patient population with, however, only moderately increased baseline creatinine prior to CTPA. This observation contrasted a number of factors in these patients, such as these patients suffering from or being prone to acute infections, being immunocompromised, some having received allogeneic transplants and being treated with cyclosporine, all of which increase the risk for renal failure [29;30]. These results are supported by previous findings questioning the causation of acute kidney injury by intravenous contrast media administration in a pivotal analysis encompassing more than 20.000 patients which found no excess risk of CIN, dialysis, or death among patients with comorbidities previously reported to predispose to nephrotoxicity^12^.

Whether the phenomenon of vessel occlusion is specific for aspergillosis (the most frequent IMD) or a sign of invasive pulmonary mold disease in general, cannot currently be revealed. Among the five proven cases according to EORTC/MSG definition, Stanzani’s cohort included one case of proven mucormycosis who was also positive for VOS. This side-finding supports preclinical data suggesting that *Mucor* spp. may also have angioinvasive properties^24^ and warrants studies similar to ours at centers with higher rates of mucormycosis. Although even rarer invasive pulmonary mold infections caused by *Zygomycetes* or *Fusarium* spp. may become more frequent in the future^25^, and despite a broad range in incidences based on local flora^26^, *Aspergillus* spp. still accounts for more than 90% of invasive human mold infections^27^, substantiating the finding of our study. In either case, the results of this study should apply to the majority of immunocompromised patients with suspected invasive pulmonary mold disease, regardless of whether the diagnostic value of CTPA is pathogen- or species-specific.

Our study has several potential limitations that have to be acknowledged. First, due to the retrospective monocentric design, a selection bias is possible. The prolonged timeframe of the study - with possible changes in patient management and procedures - might have indirectly affected the results. Although CTPA techniques could have varied during the observation period, it appears however, difficult to conceive a direction of bias due to this variation, furthermore the decision to perform CTPA instead of non-contrast enhanced CT was left at the radiologists discretion, possibly representing a confounder. As we retrospectively screened for patients with IPA, we did not specifically focus on populations with other defined infectious etiologies and therefore cannot rule out that other pathogens also display VOS.

The number of 78 proven/probable IPA patients with available CTPA may seem rather small given the long timeframe of the study; this is explained by current clinical practice, wherein the standard diagnostic recommendation is a non-contrast enhanced chest CT in most guidelines concerning immunocompromised patients^2, 28^. In addition, as the control group had to be equally immunocompromised it is probable that some of these control patients did nonetheless suffer from IPA, but could ultimately, not fulfill the very strict EORTC/MSG or Blot criteria for severe immunosuppression. Indeed, all four VOS-positive patients in the control group did show some form of microbiological evidence of IA (GM in Serum or BAL), but had however also evidence of other infections (invasive Candidiasis, *A. baumannii, C. freundii*). Thus, the diagnostic performance of VOS in our study might have been (negatively) impacted. Finally, as an inherent limitation of CTPA it should be mentioned that small lesions with a diameter ≤ 10 mm are not adequately evaluable for the presence of the VOS^22^.

In conclusion, this study confirmed that detection of VOS by CTPA in immunocompromised patients is highly suggestive of IPA and that this observation is independent of hematologic malignancies as opposed to other immunosuppressing underlying diseases. Thus, CTPA studies are likely to have additional diagnostic value over non-contrast enhanced chest CT in immunocompromised patients with suspected IPA. In addition, our data suggests that CTPA is safe with regard to a possible induction of CIN in these patients.

Therefore, this study encourages clinicians to perform CTPA on an individual basis if IPA in immunocompromised patients is suspected and diagnosis is otherwise uncertain. However, before this procedure can become a general recommendation, it needs to be validated prospectively, preferably in a multicentric trial.
